# Liposomal Formulations for Efficient Delivery of a Novel, Highly Potent Pyrimidine-Based Anticancer Drug

**DOI:** 10.3390/ph18081210

**Published:** 2025-08-15

**Authors:** Sofia Teixeira, Débora Ferreira, Ana Rita O. Rodrigues, Ligia R. Rodrigues, Elisabete M. S. Castanheira, Maria Alice Carvalho

**Affiliations:** 1 Chemistry Centre of University of Minho (CQ-UM), Campus de Gualtar, 4710-057 Braga, Portugal; id9191@alunos.uminho.pt; 2 Physics Centre of Minho and Porto Universities (CF-UM-UP), LaPMET (Laboratory of Physics for Materials and Emergent Technologies), University of Minho, Campus de Gualtar, 4710-057 Braga, Portugal; 3 Centre of Biological Engineering (CEB), University of Minho, Campus de Gualtar, 4710-057 Braga, Portugal; 4 LABBELS—Associated Laboratory, Campus de Azurém, 4800-058 Guimarães, Portugal

**Keywords:** liposomes, pyrimido[5,4-*d*]pyrimidine, anticancer activity, colorectal cancer, triple-negative breast cancer

## Abstract

**Background/Objectives:** Cancer is one of the deadliest diseases worldwide. Despite the existing treatments, the adverse side effects and the increasing drug resistance to the current therapies lead to a reduced quality of life for patients and poor prognosis. The pyrimido[5,4-*d*]pyrimidine compound (**PP**) was identified as a promising new anticancer drug due to its potent activity against colorectal and triple-negative breast cancers; however it showed poor aqueous solubility and safety profile. This study aimed the synthesis of compound **PP**, its encapsulation in liposomal formulations based on phosphatidylcholines (PC), the characterization of liposomal formulations and its biological evaluation. **Methods:** A new synthesis method for **PP** was developed. The compound was incorporated into different liposomal formulations. The hydrodynamic size, polydispersity, and zeta potential of loaded and non-loaded formulations were measured by DLS. The cytotoxic effects of compound **PP**, placebo nanoformulations, and **PP**-loaded nanoformulations were assessed in colorectal (HCT 116) and triple-negative breast cancer (MDA-MB-231) cell lines, as well as in non-tumor BJ-5ta cells. **Results:** The **PP** compound was efficiently synthesized. The **PP**-loaded liposomal formulations exhibit sizes below 150 nm, low polydispersity, and long-time stability upon storage at 4 °C. The antitumor compound was encapsulated with excellent efficiency, and sustained release profiles were obtained. The **PP** compound showed high activity against HCT 116 (IC_50_ = 2.04 ± 0.45 µM) and MDA-MB-231 (IC_50_ = 5.24 ± 0.24 µM) cell lines. DPPC-containing formulations were effective against cancer cells, but showed toxicity comparable to free **PP** in BJ-5ta normal cells. Conversely, **PP-EggPC-Chol-L** formulation displayed strong anticancer activity with residual toxicity to normal cells. **Conclusions:** The **PP**-loaded liposomal formulation, composed of 70% PC from egg yolk (EggPC) and 30% cholesterol (Chol), designated as **PP-EggPC-Chol-L**, was the most promising formulation, showing effective anticancer activity in both cancer cell lines and a significant improvement in the safety profile which is of utmost importance to progress to the next phase of drug development.

## 1. Introduction

Cancer is a disease caused by the uncontrolled growth of abnormal cells and genome mutations, which can disseminate to other parts of the body, leading to metastasis [1]. It is one of the highest threats to humankind worldwide, accounting for 10 million deaths in 2020. Among the conventional cancer treatments, such as surgery, radiation, and chemotherapy, chemotherapy is widely used, sometimes in combination with surgery or radiation. One of the common drugs used for treatment is 5-Fluorouracil (5-FU), which may be used alone or in combination with other chemotherapeutic drugs, e.g., 5-FU with oxaliplatin (FOLFOX) or 5-FU with irinotecan (FOXFIRI) [2,3,4]. However, challenges such as increased drug resistance, severe side effects, unsatisfactory response rates, and low tumor-specific selectivity contribute to a reduced quality of life for patients and poor prognosis [2,3]. To overcome these concerns, several targeted therapies have been explored. These approaches use small molecules, antibodies, or a combination of both to specifically target cancer cells, preventing tumor growth, cell proliferation, differentiation, and migration [5,6,7]. Moreover, novel strategies have been developed to combat cancer, including advanced targeted therapies [7,8,9,10], innovative radiotherapy approaches [11,12], new nanomedicines [13,14] and emerging therapeutic agents [15,16].

**Figure 1:**
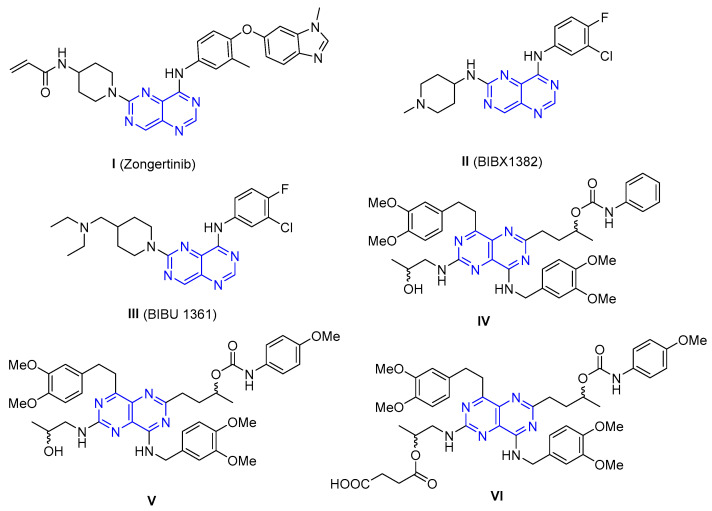
Pyrimido[5,4-*d*]pyrimidines with anticancer activity.

Compounds based on pyrimido[5,4-*d*]pyrimidine scaffold (in blue), exemplified in Figure 1, have been presented as targeted anticancer agents, whose may inhibit signaling pathways by either directly inhibiting tyrosine kinase receptors, like EGFR and HER2, or inhibiting nucleoside-nucleobase transport, or by inhibiting signaling pathways, like Akt/mTOR (protein kinase B/mammalian target of rapamycin) and Janus kinase/signal transducer and activator of transcription 3 (JAK/STAT3) [17,18,19].

In HER2-driven tumors, compound **I** (Zongertini), a covalent selective HER2 inhibitor, demonstrated strong activity by inhibiting tumor growth. Moreover, in early phase clinical trials this compound showed promising tumor responses in cholangiocarcinoma and breast cancer patients [17]. Compounds **II** (BIBX 1382) and **III** (BIBU 1361) exhibited nanomolar range activity as EGFR tyrosine kinase inhibitors. By inhibiting tumor growth, both EGFR inhibitors demonstrated anticancer efficacy [18,19]. Additionally, compound **III** (BIBU 1361) reduced levels of the pro-inflammatory cytokine IL-6 and inhibited the Akt/mTOR and gp130/JAK/STAT3 pathways [18].

Nevertheless, compounds **IV**, **V**, and **VI** exhibited potent nucleoside-nucleobase transport inhibition in lung cancer cells, COR-L23 cells, with IC_50_ values of 8, 4, and 7 nM, respectively [20].

Given that pyrimido[5,4-*d*]pyrimidine core offers a rigid, planar scaffold ideal for antitumor agents, a new derivative having a phenyl hydrazide unit at position 4 and a 4-CF_3_OC_6_H_4_NH unit at position 8 of the heterocycle was designed. It is known that the presence of OCF_3_ group improves metabolic stability and membrane permeability [21]. However, the hydrophobic nature of this group may hinder aqueous solubility, necessitating delivery strategies such as liposomal encapsulation to improve bioavailability and targeted delivery. The NH groups enable hydrogen bonding and precise binding to oncogenic targets while amide–phenyl linkage adds rigidity, hydrogen-bonding, and hydrophobic/π–π interactions typical of kinase inhibitors [22]. The idealized compound **PP** was synthesized which showed great activity against cancer cell line HCT 116 [23].

During the process of drug discovery and development, aqueous solubility issues and toxicity often arise while trying to increase the potency of the new compounds, preventing their progress in the pipeline [24]. This was the case of the newly synthesized pyrimido[5,4-*d*]pyrimidine compound (**PP**) which exhibited excellent in vitro anticancer activity but displayed poor solubility in aqueous medium and low safety profile. To tackle these issues, nanoformulations have been proposed to enhance drug solubility and overcome pharmacokinetics and toxicity challenges [24,25,26]. Liposomes have been used to deliver poorly water-soluble drugs, and several formulations were approved by the U.S. FDA for cancer therapeutics [27,28]. Their structural similarity to cell membranes enables the efficient delivery of bioactive compounds [29]. Liposomal nanoformulations easily incorporate hydrophobic drugs into their phospholipid bilayer [30] and are known for their good bioavailability, biocompatibility, and biodegradability [31,32]. Here, main phospholipid components of the biological membranes (phosphatidylcholines) were chosen for liposomal nanocarriers, with different fluidities, also modulated by the presence of cholesterol [33]. While DPPC is rigid at room temperature (gel phase), unsaturated phosphatidylcholines from natural sources (egg yolk, soybean) form more permeable and fluid liposomes [34]. Liposomes of egg lecithin with 30% of cholesterol have also been employed as models in studies of membrane permeation [35].

Liposomal systems encapsulating of pyrimido[5,4-*d*]pyrimidine derivatives have not yet been described. Therefore, in this work we present the synthesis method to generate the new PP, the developed liposomal formulations of the new compound **PP** to improve its limited solubility in water, and their anticancer activity in vitro against colorectal (HCT 116) and triple-negative breast (MDA-MB-231) cancer cells.

## 2. Results and Discussion

### 2.1. Synthesis of Hit Drug PP

**Scheme 1:**
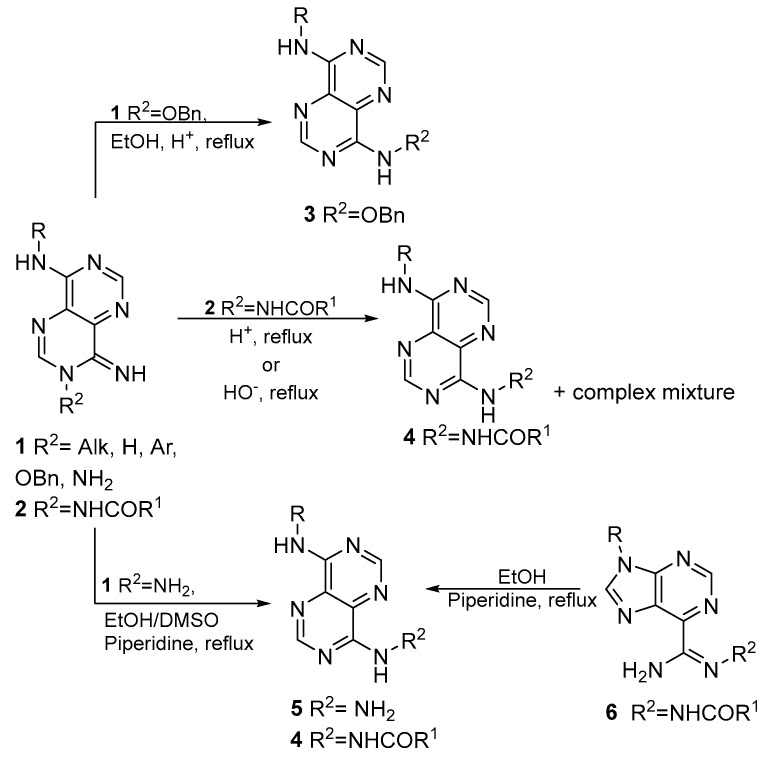
Methods of synthesis of pyrimido[5,4-*d*]pyrimidines.

In literature, Monier et al. [36] highlighted synthetic approaches to generate pyrimido[5,4-*d*]pyrimidines and their high pharmacological importance as anticancer, antioxidant, antidiabetic, anti-*Mycobacterium tuberculosis*, and antibacterial agents. These derivatives were often synthesized from tetrachloride pyrimido[5,4-*d*]pyrimidine structures through chlorine substitution with nucleophiles [37,38,39]. Another synthesis method includes the reaction of acyclic diaminomaleonitrile derivatives with aldehydes [40,41] and the nucleophilic addition of amines to purine derivatives [39,42,43]. Additionally, the Dimroth rearrangement’s reaction could be used to convert compounds **1** or **2** into **3** or **4** (Scheme 1), respectively, refluxing compounds **1** or **2** under acidic or basic conditions [44]. In our research group, compounds **1** (R^2^ = OBn) were refluxed under acid catalysis yielding the desired product **3** [45]. However, when compounds **2** (R^2^ = NHCOR^1^) were refluxed in acid or basic ethanolic medium, compounds **4** were isolated as the major component of complex mixtures [46]. Besides, a new method was developed, converting 6-amidinopurines **6** into pyrimido[5,4-*d*]pyrimidines **4** after piperidine treatment, under heat [46]. Recently the products **5** (R^2^ = NH_2_) were also obtained from the respective precursors **1** under similar reaction conditions [47].

**Scheme 2:**
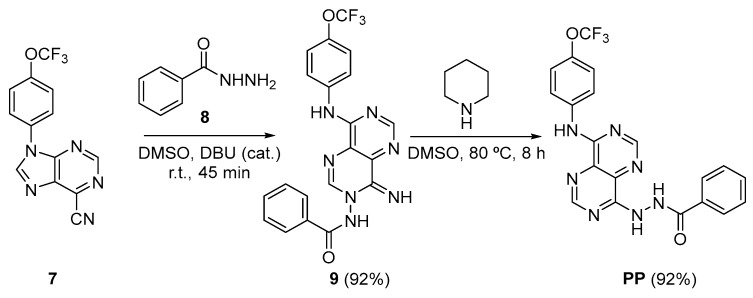
Synthesis of the hit compound **PP**.

In this work, the hit compound **PP** was synthesized following the synthetic approach depicted in Scheme 2. The 6-cyanopurine **7** and compound **9** were synthesized following methods previously reported in literature with the necessary adjustments [45,47,48,49,50,51]. Compound **7** was reacted with hydrazide **8,** in DMSO, in the presence of a catalytic amount of DBU at room temperature. The reaction was monitored by TLC and was completed after 45 min. The compound **9** was isolated in excellent yield after addition of water to the reaction mixture followed by filtration. Then, Dimroth rearrangement’s reaction conditions were applied to obtain compound **PP**. Compound **9** reacted with piperidine, in DMSO, at 80 °C. When the reaction was completed by TLC, the piperidine was removed from the reaction mixture by evaporation under reduced pressure, and water was added to precipitate the product, which was isolated in excellent yield (92%).

### 2.2. Characterization of Drug-Loaded Liposomes

Liposomes (**L**) are nanosystems that can deliver hydrophilic and hydrophobic drugs, have cellular affinity and tissue compatibility, reduce drug toxicity, and improve drug stability [30,52]. Here, liposomes of phosphatidylcholine (PC), the main component of biological membranes, were prepared, either from a natural phospholipid mixture (lecithin from egg yolk, EggPC) or using the synthetic dipalmitoylphosphatidylcholine (DPPC). These phospholipids differ in the phase transition temperature (T_c_), EggPC with a T_c_ between −5 °C and −15 °C and DPPC with a T_c_ of 41 °C. Therefore, EggPC and DPPC liposomes differ in the membrane microviscosity at room temperature and at body temperature (37 °C) [30,52,53,54]. Cholesterol (Chol) at 30% molar ratio was also used in combination with Egg-PC or DPPC lipids, with the intent of changing the fluidity of liposomes.

The Hit antitumor compound, **PP**, was incorporated into the different liposomal formulations, **PP-EggPC-L**, **PP-DPPC-L**, **PP-EggPC-Chol-L**, and **PP-DPPC-Chol-L**. The hydrodynamic size, polydispersity, and zeta potential of loaded and non-loaded formulations were measured by DLS (Table 1).

**PP-EggPC-L** and **PP-DPPC-L** showed sizes below 150 nm, and the addition of cholesterol did not affect the hydrodynamic size and polydispersity, which remains around (or below) 0.25, pointing to a narrow size distribution. As shown in Table 1, these sizes are very similar to the ones of non-loaded EggPC, Egg-PC/cholesterol, and DPPC liposomes, indicating that **PP** has a negligible influence on the nanostructures’ size. Comparing the non-loaded and the drug-loaded formulations, the PDI is maintained and the variations in zeta potential are negligible. The particles’ diameter below 200 nm favors the occurrence of enhanced permeability and the retention (EPR) effect, allowing passive accumulation through leaky vasculature [55]. These sizes also indicate that the drug-loaded nanocarriers could attain suitable systemic circulation times, avoiding rapid clearance and excessive uptake by immune cells [56,57]. Zeta-potential values indicate near neutral charge, as expected from the zwitterionic character of PC, the addition of cholesterol not influencing the surface charge. Small differences in zeta potential are detected for EggPC formulations (slightly negative) and DPPC ones (slightly positive). In DPPC, the small choline group may be more exposed, giving rise to a small positive charge. In EggPC, the lower rigidity, with more flexible hydrocarbon tails, can promote the phosphate group to be more exposed at the surface, originating a slightly negative charge.

**Figure 2:**
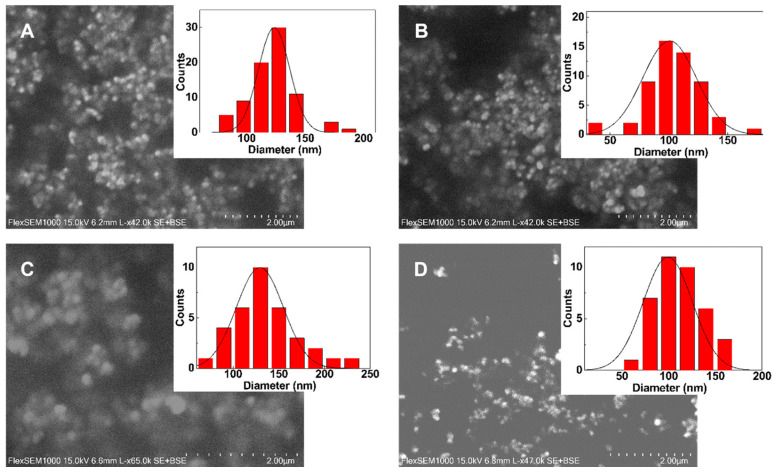
SEM images of drug-loaded liposomal formulations and corresponding size histograms: **PP-EggPC-L** (**A**), **PP-DPPC-L** (**B**), **PP-EggPC-Chol-L** (**C**), and **PP-DPPC-Chol-L** (**D**).

Scanning Electron Microscopy images evidence roughly spherical nanostructures, with sizes in accordance with DLS results (considering the application of vacuum in SEM measurements), as observed in the size histograms, fitted to a Gaussian distribution (Figure 2). **PP-EggPC-L** exhibit sizes of 126 ± 17 nm (Figure 2A), while **PP-DPPC-L** display particles with 101 ± 17 nm diameter (Figure 2B). Structures containing cholesterol (Figure 2C,D) show no appreciable influence of the presence of cholesterol in the mean size. **PP-EggPC-Chol-L** (Figure 2C) show structures with sizes of 129 ± 26 nm, while **PP-DPPC-Chol-L** (Figure 2D) evidence smaller particles, with diameters of 99 ± 22 nm.

**Figure 3:**
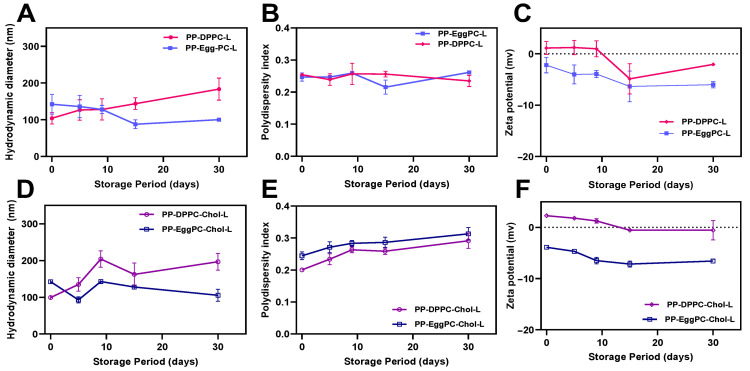
Assay of stability, at 25 °C, of liposomal formulations **PP-EggPC-L** and **PP-DPPC-L**, based on liposome size (**A**), size distribution (**B**), and zeta potential (**C**), and assay of stability of liposomal formulations **PP-EggPC-Chol-L** and **PP-DPPC-Chol-L** based on liposome size (**D**), size distribution (**E**), and zeta potential (**F**), for 30 days after storage at 4 °C. The stability of the formulations is compared to the original values (measured at day 0). Results presented as mean and standard deviation (SD) of replicate assays.

The stability of drug-loaded liposomal formulations was for 30 days, measuring the variation of particles’ size, size distribution, and zeta potential at room temperature (Figure 3). The storage was performed at 4 °C, which is the most common storage temperature for lipid vesicles in aqueous media [58]. The stability of lipid systems depends on their molecular composition (e.g., lipid chain length), physical state (transition temperature), and surface charge [58,59,60,61]. It has been found that longer acyl chains increase the stability of lipid bilayers [62], causing stronger van der Waals interactions, with a corresponding increase in the transition temperature [63]. All formulations have shown minor variations in size, PDI, and zeta potential until 10 days of storage. After 10 days of storage, it is clear that the size of **PP-EggPC-L** and **PP-DPPC-L** had a small decrease and increase, respectively, until 30 days of storage. While DPPC liposomes have a slight tendency for aggregation over time, the fluid EggPC formulation shows a size decrease, pointing to some compaction of the lipid chains (that can be induced by the hydrophobic drug). Also, the zeta potential of **PP-DPPC-L** turned slightly negative (but near zero) over time.

Besides changing the fluidity of **PP-EggPC-L** and **PP-DPPC-L** formulations, adding cholesterol could improve the stability of the formulations [64]. As shown in Figure 3, **PP-EggPC-Chol-L** and **PP-DPPC-Chol-L** nanoformulations showed similar variations in hydrodynamic size, PDI, or zeta potential over time when compared to **PP-EggPC-L** and **PP-DPPC-L** liposomes, respectively. Moreover, both **PP-DPPC-Chol-L** and **PP-EggPC-Chol-L** formulations present a similar behavior in the zeta-potential plot (Figure 3F), wherein the charge became more negative around day 15, identical to the formulations without cholesterol, but still maintaining near neutral charge. Therefore, all Egg-PC and DPPC formulations have shown great stability.

One common method to extend the storage period of liposomes is freeze-drying, which is generally applied to increase shelf stability of liposomal formulations [64,65]. Here, the formulations were lyophilized and then resuspended in sterile water at a concentration five times higher, and the hydrodynamic diameter and PDI were assessed after resuspension of the nanoformulations (Table 2). The formulations showed small changes in size values and PDI < 0.3, with exception of **PP-DPPC-L** and **PP-EggPC-Chol-L** which showed a higher hydrodynamic diameter. So, considerable aggregation was verified for the latter formulations, and in further studies a cryo-protectant should be used to minimize the aggregation.

### 2.3. Drug Encapsulation Efficiency and Release Profiles

The determination of the encapsulation efficiency (EE%) of the drug **PP** in nanoformulations and the release profiles to aqueous media were assessed by fluorescence spectroscopy. From the UV/Vis absorption spectrum (Figure S1A), an excitation wavelength of λ_exc_ = 400 nm was used for the fluorescence emission and the determination of the calibration curve. The obtained encapsulation efficiencies (determined by Equation (1)) of **PP** in the several formulations were excellent (Table 3).

**Figure 4:**
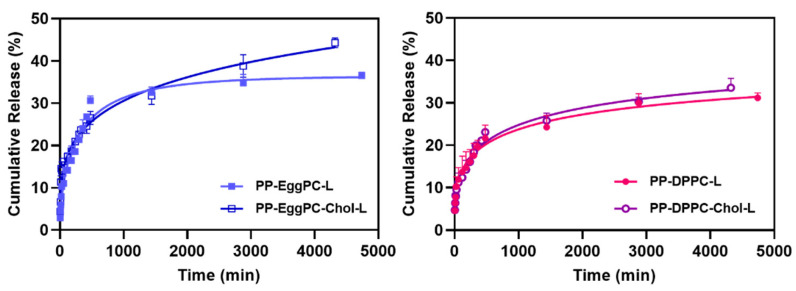
Cumulative release of the antitumor compound from **PP-EggPC-L**, **PP-EggPC-Chol-L**, **PP-DPPC-L**, and **PP-DPPC-Chol-L** liposomes, fitted to the Weibull model. Results presented as mean and standard deviation of replicate assays.

Release assays of the antitumor compound from the different formulations to aqueous media were carried out, for 72 or 79 h. After 24 h, the cumulative release percentage (around 25%) from DPPC-based formulations was lower than for EggPC ones (~30%), which may be due to the higher microviscosity of DPPC liposomes. The release from **PP-EggPC-L** and **PP-EggPC-Chol-L** (Figure 4) was similar in the first 30 h, and the same result was verified for DPPC-based formulations (Figure 4). However, the **PP-EggPC-Chol-L** formulation ensures a higher drug release for 72 h, showing a benefit from the addition of cholesterol.

The release profiles were fitted to the first-order kinetic model [66] (Equation (2)) and to the Weibull model [67] (Equation (3)) and the results are displayed in Table S1 in Supplementary Materials. The fitting to the first-order kinetic model is generally poor. The Weibull model is more suitable to fit to the release profile data (Figure 4), with high coefficients of determination for all liposomal formulations. The release mechanism of the liposomes seems to be dominated by Fickian diffusion (*b* values equal or below 0.75) [67], meaning a process determined by the concentration gradient.

### 2.4. Anticancer Activity

The cytotoxicity of the synthesized drug **PP** and all **PP**-loaded liposomal nanoformulations was evaluated in two different cancer cell lines: one representative of colorectal cancer (HCT 116), a disease of particular relevance since it is the second leading cause of cancer-related deaths worldwide [68], and the triple-negative breast cancer MDA-MB-231 cells, the most aggressive breast cancer subtype with poor prognosis, primarily due to its propensity to metastasize to various organs [2]. The toxicity of **PP** and **PP**-loaded nanoformulations was also assessed in BJ-5ta normal cells. 5-FU was used as the experimental control.

**Figure 5:**
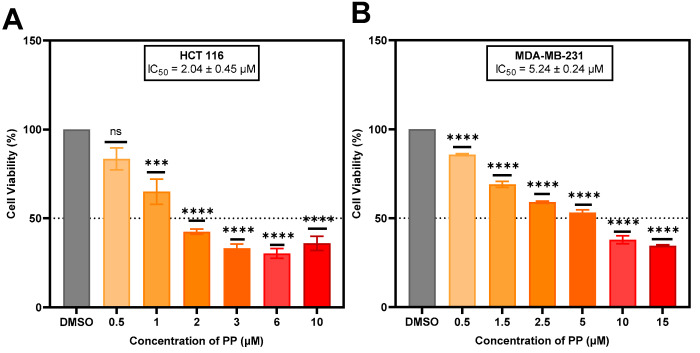
Assessment of the viability of HCT 116 (**A**) and MDA-MB-231 (**B**) cancer cells after a 48 h exposure to **PP** over a 48 h treatment period. HCT 116 (**A**) was exposed to **PP** concentrations of 0.5, 1, 2, 3, 6, and 10 µM and **MDA-MB-231** (**B**) to **PP** concentrations of 0.5, 1.5, 2.5, 5, 10, and 15 µM. Cell viability was determined using the MTT colorimetric assay and normalized to DMSO-treated cells. The resulting data are presented as IC_50_ (in µM) ± SEM (standard error of the mean) from three independent experiments. One-way ANOVA indicates statistically significant differences to DMSO assessed by Dunnett’s post-test and was denoted as follows: ns (non-statistical significance), *** *p* < 0.001, and **** *p* < 0.0001.

In HCT 116 and MDA-MB-231 cell lines, cell viability decreased in a dose-dependent manner with increasing **PP** concentrations (Figure 5). The cytotoxic activity corresponds to IC_50_ values of 2.04 ± 0.45 µM for HCT 116 and 5.24 ± 0.24 µM for MDA-MB-231 cells. Notably, **PP** exhibited significantly higher activity compared to 5-FU (control), which showed IC_50_ values of 10.39 ± 0.68 µM and 183.49 ± 17.13 µM for the respective cell lines (Figure S2 in Supplementary Materials).

Before assessing the activity of **PP**-loaded liposomal nanoformulations, the cytotoxicity of placebo (non-loaded) liposomes was evaluated at lipid concentrations of 0.190 mM or 0.375 mM for HCT 116 and 0.314 mM or 0.943 mM for MDA-MB-231 cells. According to ISO 10993-5, cell viability above 70% in MTT assays indicates non-cytotoxicity. Overall, placebo liposomal formulations showed no significant toxicity. However, **DPPC-Chol-L** had a viability close to 70% in HCT 116 cells, while **DPPC-L** exhibited higher cytotoxicity (viability < 70%) in MDA-MB-231 cells (Figure S3 in Supplementary Materials). Lipid-based formulations are usually regarded as biocompatible and non-toxic [69]; however, the rigid nature of certain lipids, such as DPPC, may cause membrane disruption, leading to cytotoxic effects on cancer cells. It must be referred that this may be a specific effect of our drug-loaded nanosystems and/or the cells types used.

**Figure 6:**
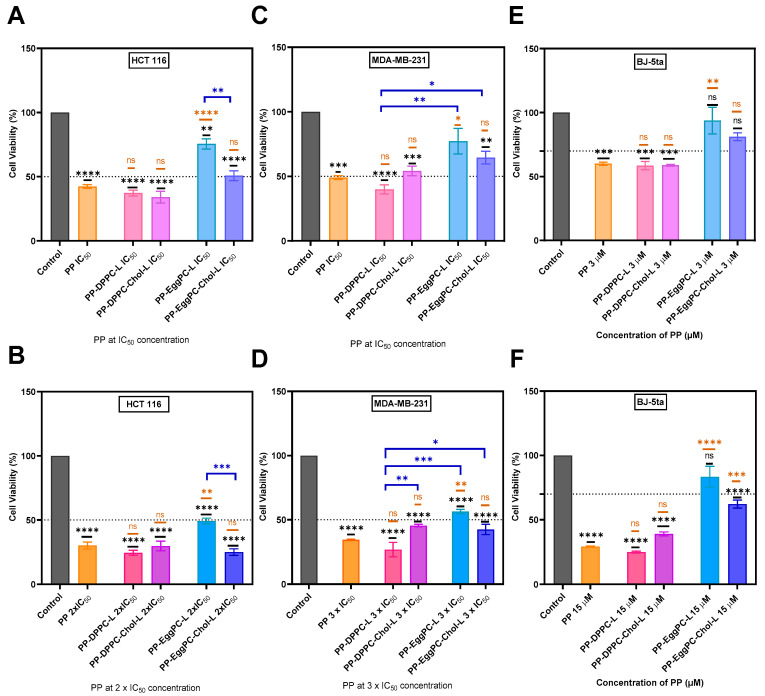
Assessment of the viability of HCT 116 (**A**,**B**), MDA-MB-231 (**C**,**D**), and BJ-5ta (**E**,**F**) after a 48 h exposure to drug-loaded liposomal nanoformulations. HCT 116 cells were exposed to **PP** and drug-liposomal nanoformulations at **PP** IC_50_ (**A**) and a 2-fold IC_50_ concentration (**B**). MDA-MB-231 cells were exposed to **PP** and drug-liposomal nanoformulations at **PP** IC_50_ (**C**) and a 3-fold IC_50_ concentration (**D**). BJ-5ta cells were exposed to **PP** and drug-liposomal nanoformulations at **PP** concentrations of 3 µM (**E**) and 15 µM (**F**). Cell viability was determined using the MTT colorimetric assay. The cell viability of **PP** was normalized to DMSO, while drug-liposomal-based nanoformulations were normalized to untreated cells. One-way ANOVA indicates statistically significant differences within the group assessed by Tukey’s post-hoc test and was denoted as follows: ns (non-statistical significance), *p* > 0.05, * *p* ≤ 0.05, ** *p* ≤ 0.01, *** *p* < 0.001, and **** *p* < 0.0001. Black * represents statistical significance of **PP** or **PP**-loaded formulations relative to control. Orange * represents statistical significance of **PP**-drug loaded formulations to the **PP** drug. Blue * represents statistical significance between **PP**-loaded formulations.

The drug-loaded nanoformulations were tested at IC_50_, 2-fold IC_50_, and 3-fold IC_50_ in both cancer cell lines and compared to the free drug. The assays were performed based on a compound IC_50_ value in each cell line. The multiples of IC_50_ concentrations were used to verify if the response was proportional to the increase in compound concentration in each cell line. As shown in Figure 6A–D, all liposomal formulations, apart from **PP-EggPC-L**, showed no statistical significance (*p* > 0.05) when compared to **PP** at different concentrations, in both cancer cell lines.

It was verified that lipid composition of the formulations influenced the cell viability in HCT 116 and MDA-MB-231 cells. Differences were identified between **PP-EggPC-L** and **PP-EggPC-Chol-L**, and occasionally between **PP-DPPC-L** and **PP-DPPC-Chol-L** (Figure 6A–D). These variations may stem from cholesterol’s effect on membrane viscosity, which depends on phospholipid type and temperature. Cholesterol generally acts as a buffer for membrane fluidity, making membranes less fluid in liquid-crystalline phases and more fluid in the gel phase [33,70,71]. EggPC, rich in unsaturated phospholipids, has a more disordered structure compared to saturated lipids. The unsaturated bonds introduce kinks that result in greater spacing and higher fluidity at physiological temperatures. At low concentrations, cholesterol intercalates between phospholipid molecules, reducing EggPC membrane’s fluidity by restricting the motion of fatty acids’ acyl chains [71,72]. In contrast, DPPC (16:0 PC) is a saturated phospholipid that forms an ordered gel-like state at room temperature, with a phase transition (T_c_) around 41 °C [73], above which the liquid-crystalline phase is attained. Below T_c_, cholesterol disrupts the ordered packing of DPPC, introducing disorder within the tightly packed acyl chains, with a corresponding increase in membrane fluidity [33,74].

In HCT 116 cells (Figure 6A,B), **PP**-loaded liposomal formulations with rigid lipids (**PP-DPPC-L**) showed greater activity, whereas those with fluid lipids (**PP-EggPC-L**) exhibited lower activity. Adding cholesterol to DPPC liposomes had little effect. However, in contrast, its incorporation into the Egg-PC formulation significantly increased antitumor activity compared to **PP-EggPC-L** (*p* ≤ 0.01 at IC_50_ concentration and *p* < 0.0001 at 2 × IC_50_).

In MDA-MB-231 cells (Figure 6C,D), **PP-EggPC-L** showed the lowest activity, similar to HCT 116 cells. Furthermore, **PP-DPPC-Chol-L** formulations showed lower activity than **PP-DPPC-L**, with statistical significance (*p* ≤ 0.01) at a 3-fold IC_50_ concentration. **PP-DPPC-L**, the most rigid formulation, exhibited the highest activity, significantly outperforming **PP**-EggPC- (*p* ≤ 0.01 at IC_50_ concentration and *p* < 0.001 at 3 × IC_50_) and **PP**-EggPC-Chol- (*p* ≤ 0.05 at IC_50_ and 3 × IC_50_) based formulations.

Overall, **PP-DPPC-L** and **PP-DPPC-Chol-L** formulations demonstrated superior activity compared to **PP-EggPC-L** at both concentrations and for both cell lines. Although **PP-EggPC-L** displayed the lowest activity among the formulations, compared to free drug, its activity significantly improved upon cholesterol addition.

Afterward, the toxicity of free **PP** and drug-loaded formulations was tested against non-tumor BJ-5ta cells at **PP** different concentrations (3 µM and 15 µM). The concentrations used for normal cells were based on a lower concentration of 3 µM, which is in between the IC_50_ of both cancer cell lines (2 and 5 µM) and the highest concentration tested, 15 µM (3 × IC_50_ of **PP** in MDA-MB-231 cell line).

At 3 µM (Figure 6E), drug-loaded formulations showed varying toxicity-based lipid composition. **PP-DPPC-L** and **PP-DPPC-Chol-L** exhibited cytotoxicity levels comparable to the free drug, while **PP-EggPC-L** and **PP-EggPC-Chol-L** showed lower toxicity, with no statistically significant differences compared to the control. Cell viability remained above 70%, indicating non-toxicity at this concentration.

At 15 µM, increasing the **PP** concentration resulted in higher toxicity (Figure 6F). **PP-DPPC-L** and **PP-DPPC-Chol-L** formulations showed toxicity levels comparable to the free drug. **PP-EggPC-L** showed no toxicity to normal cells, while **PP-EggPC-Chol-L** demonstrated low toxicity. These EggPC-based formulations significantly reduced drug toxicity when compared to free **PP** (**PP-EggPC-L** showed *p* ≤ 0.01 at 3 µM and *p* < 0.0001 at 15 µM; **PP-EggPC-Chol-L** showed *p* < 0.001 at 15 µM).

In summary, DPPC-containing formulations were effective against cancer cells but showed toxicity comparable to free **PP** in BJ-5ta normal cells. Conversely, **PP-EggPC-Chol-L** formulation was the one that displayed strong anticancer activity with residual toxicity to normal cells, making it the safest formulation, truly improving the therapeutic window. This aligns with previous reports showing that nanoformulations enhance drug safety profiles [24]. Future research should focus on functionalizing **PP-EggPC-Chol-L** to target overexpressed cancer cell receptors, enhancing its therapeutic potential.

**Figure 7:**
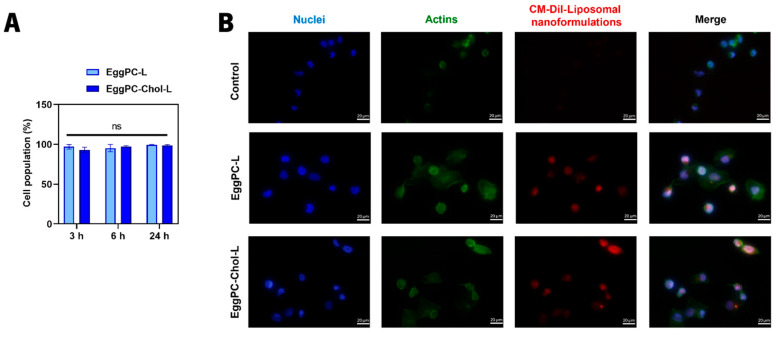
Assessment of MDA-MB-231 cellular internalization of CM-DiI-labeled liposomal formulations by flow cytometry (**A**) and confocal laser scanning microscopy (**B**). (**A**) Quantitative analysis of cells treated with **EggPC-L** and **EggPC-Chol-L**, stained with CM-DiI and measured by flow cytometry. Data are presented as the percentage of cells ± SD of three independent experiments. ns: non-statistical significance. (**B**) MDA-MB-231 cellular internalization of CM-DiI-labeled **EggPC-L** and **EggPC-Chol-L**, 24 h after administration, by microscopy. Liposomal formulations were labeled with CM-DiI (red), nuclei stained with DAPI (blue), and actins with Alexa Fluor-488 phalloidin (green). Scale bar: 20 µm.

To evaluate whether liposome internalization and intracellular targeting impacted their activity, uptake experiments were conducted. **EggPC-L** and **EggPC-Chol-L** were selected due to their shared phospholipid composition but differing membrane rigidity. CM-DiI labeling allowed quantification and visualization in MDA-MB-231 cells. As illustrated in Figure 7A, flow cytometry revealed a high uptake of both formulations, with over 93% of cells internalizing liposomes within 3 h, increasing to approximately 99% at 24 h. These results confirm successful internalization, suggesting that membrane rigidity does not significantly impact the uptake efficiency.

Additionally, microscopy further confirmed cellular uptake in MDA-MB-231 cells (Figure 7B), showing clear internal localization of both liposomal formulations. Strong red fluorescence was observed upon incubation with CM-Dil-labeled liposomal formulations, mostly in the nuclei rather than in the actins. Previous studies [75] suggest that rigid liposomal formulations exhibit higher anticancer activity due to better internalization compared to fluid ones. However, our results show similar internalization of both fluid and rigid liposomes in MDA-MB-231 cells. In fact, the main component of both formulations is the same (100% EggPC or 70% EggPC), and this effect may also be cell-dependent. Nevertheless, these findings indicate that both formulations are successfully internalized in aggressive MDA-MB-231 triple-negative breast cancer cells, known for their high growth rates, metastatic ability and drug resistance [76].

## 3. Materials and Methods

### 3.1. Chemistry

The materials included the following: Solvents (Fisher) and other chemicals commercially available (Sigma-Aldrich, St. Louis, MO, USA) were used as shipped, unless mentioned in the experimental protocol. The melting points were determined with a Gallenkamp melting point apparatus and are uncorrected. The reactions were monitored by thin layer chromatography (TLC) using Silica Gel 60 F_254_ (Merck, Darmstadt, Germany) with detection by UV light. The NMR spectra were recorded on a Bruker Avance III NMR spectrometer (^1^H: 400 MHz; ^13^C: 100 MHz) including the ^1^H and ^13^C correlation spectra (HMQC and HMBC), for solutions in DMSO-*d*_6_ at 298 K. Chemical shifts (*δ*) were reported in parts per million (ppm), and the coupling constants, *J*, were reported in hertz (Hz). The purities of the tested compound were higher than 95% by elemental analysis, which were reported to be within 0.4% of calculated values. IR spectra were recorded with a Spectrum Two, PerkinElmer in mode ATR. Elemental analyses were performed with a LECO TruSpecMicro CHNS instrument.

#### 3.1.1. Synthesis of *N*-(4-Imino-8-((4-(trifluoromethoxy)phenyl)amino)pyrimido[5,4-*d*]pyrimidin-3(4*H*)-yl)benzamide **9**

To a suspension of 6-cyanopurine **7** (0.100 g, 0.329 mmol) and 1.5 equivalents of hydrazide **8** (0.069 g, 0.049 mmol), in DMSO, a catalytic amount of DBU was added under magnetic stirring. The reaction was followed by TLC, and after 45 min this showed the absence of limiting reagent, so distilled water was added to the reaction mixture. The solid in suspension was filtrated and washed with distilled water, and pyrimidopyrimidine **9** (0.134 g, 0.302 mmol, 92%) was obtained as a white solid: m.p. 272 °C, dec; ^1^H NMR (DMSO-*d*_6_, 400 MHz): δ 11.0–8.9 (br. s, 3H, NH), 8.69 (s, 1H), 8.58 (s, 1H), 8.12 (d, *J* = 7.2 Hz, 2H), 8.07 (d, *J* = 8.8 Hz, 2H), 7.50 (d, *J* = 7.2 Hz, 1H), 7.44 (t, *J* = 7.2 Hz, 2H), and 7.36 (d, *J* = 8.8 Hz, 2H); ^13^C NMR (DMSO-*d*_6_, 100 MHz): δ 168.18, 156.37, 155.25, 152.69, 148.57, 144.12, 144.11, 137.19, 135.37, 130.24, 127.79, 127.42, 126.19, 123.21, 120.78, and 123.73–116.10 (q, *J* = 254 Hz, OCF_3_); IR *ν_max_* (cm^−1^): 3500–2900 (br), 1661, 1616, 1580, 1558, 1564, 1526, and 1506; Anal. Calcd for C_20_H_14_F_3_N_7_O_2_.0.8H_2_O: C: 52.70; H: 3.45; N: 21.51; and found: C: 52.62; H: 3.44; and N: 21.40.

#### 3.1.2. Synthesis of *N*’-(8-((4-(Trifluoromethoxy)phenyl)amino)pyrimido[5,4-*d*]pyrimidin-4-yl)benzohydrazide **PP**

To a suspension of pyrimidopyrimidine **9** (0.087 g, 0.197 mmol) in DMSO (0.3 mL), 2 equivalents of piperidine were added. The reaction mixture was placed under magnetic stirring, at 80 °C, and the reaction was followed by TLC. After 8 h, it showed the absence of reagent **9**, and piperidine was eliminated by using the rotary evaporator, under reduced pressure. Then, water was added, and the resulting suspension was filtrated and washed with water and ethanol, to obtain product **PP** (0.080 g, 0.182 mmol, 92%) as a yellow solid: m.p. 253 °C (dec); ^1^H NMR (DMSO-*d*_6_, 400 MHz): δ 10.71 (s, 1H, NH), 10.39 (s, 1H, NH), 10.33 (s, 1H, NH), 8.70 (s, 1H), 8.63 (s, 1H), 8.15 (d, *J* = 8.4 Hz, 2H), 7.96 (d, *J* = 7.2 Hz, 2H), 7.61 (d, *J* = 7.2 Hz, 1H), 7.53 (t, *J* = 7.2 Hz, 2H), and 7.40 (d, *J* = 8.4 Hz, 2H); ^13^C NMR (DMSO-*d*_6_, 100 MHz): δ 165.48, 159.02, 156.49, 154.69, 153.99, 144.00, 137.71, 132.48, 132.06, 131.90, 131.22, 128.49, 127.54, 123.14, 121.29, and 123.96–116.34 (q, *J* = 254 Hz); IR *ν_max_* (cm^−1^): 3338, 3277, 3262, 3211, 3181, 3062, 1625, 1612, 1597, 1574, 1525, and 1506; Anal. Calcd for C_20_H_14_F_3_N_7_O_2_: C: 54.42; H: 3.20; N: 22.22; and found: C: 54.59; H: 3.28; and N: 22.20.

### 3.2. Liposomal Nanoformulations

#### 3.2.1. Drug-Loaded Liposome Preparation and Characterization

Liposomal formulations based on dipalmitoylphosphatidylcholine (**DPPC-L**), DPPC:cholesterol (7:3 molar ratio) (**DPPC-Chol-L**), phosphatidylcholine from egg yolk (**EggPC-L**), and EggPC:cholesterol (7:3 molar ratio) (**EggPC-Chol-L**), for in vitro testing, were prepared following the ethanol injection method [77]. The drug-loaded liposomes were prepared following the co-injection method [77], as previously described [78]. Briefly, a lipid film was prepared by evaporating 250 µL of a lipid stock solution (20 mM) with or without cholesterol (10 mM stock solution), to completely remove the solvent chloroform, and then 400 µL of **PP** (0.2 mM) in ethanol was added. The mixed solution was injected (at a rate of 200 µL/min) under vigorous vortexing, into 5 mL of a phosphate buffer solution (pH = 7.4), above the lipids’ melting transition temperature (at 55 °C in the case of DPPC). The final lipid and antitumor compound concentrations were 1 mM and 16 µM, respectively. The same formulations were prepared without any drug (placebo formulations). The nanoformulations in aqueous media were frozen at −20 °C overnight and then subjected to lyophilization in LaboGene ScanVac CoolSafe Pro −110 °C at approximately 0.8 mbar.

In uptake experiments, **EggPC-L** and **EggPC-Chol-L** formulations (5 mM of lipid) were labeled by adding 7.5 µL of a 1 mM DMSO stock solution of CM-DiI dye. The suspensions were incubated at 37 °C for 1 h, and unincorporated dye was removed using Amicon Ultra Centrifugal Filters (10 kDa, Merck, Darmstadt, Germany) at 8000× *g* for 10 min.

The average hydrodynamic size, polydispersity index, and zeta potential of the formulations (placebo and drug-loaded) in PBS buffer were measured in a Dynamic Light Scattering (DLS) equipment Litesizer™ 500 from Anton Paar (Anton Paar GmbH, Graz, Austria), using a semiconductor laser diode of 40 mW and λ = 658 nm, a backscatter angle (175°) and a controlled temperature of 25 °C. Three independent measurements were performed for each sample, and the experimental data were processed using Kalliope™ software (Anton-Paar GmbH, Graz, Austria), to obtain the distribution by intensity. The results are given as mean and corresponding standard deviation (SD).

SEM images were obtained in a Hitachi FlexSEM 1000 II microscope (Hitachi High-Tech Corporation, Tokyo, Japan), operating at 15 kV. The processing of SEM images was performed using ImageJ software (version 1.53t, National Institutes of Health (NIH), Bethesda, MD, USA), which consisted in enhancing local contrast and adjusting brightness.

#### 3.2.2. Drug Encapsulation and Release

##### Equipment

UV/Visible absorption and fluorescence spectroscopy measurements were used to determine encapsulation efficiency and release profiles of the antitumor compound. UV/visible absorption spectra were measured in a Shimadzu UV-3600 Plus UV-Vis-NIR spectrophotometer (Shimadzu Corporation, Kyoto, Japan). Fluorescence spectra were determined in a Fluorolog 3 HORIBA Jobin Yvon spectrofluorimeter (HORIBA Jobin Yvon IBH Ltd., Glasgow, UK), equipped with Glan-Thompson polarizers, double monochromators in excitation and emission, and a temperature-controlled cuvette holder. From the experimental absorption spectrum, the excitation of **PP** compound was set at λ_exc_ = 400 nm. The fluorescence emission spectra were collected between 410 nm and 650 nm, with slits of 4 nm in both excitation and emission.

##### Determination of Encapsulation Efficiency

The encapsulation efficiency, EE (%), of the antitumor compound **PP** in the obtained nanoformulations was assessed through fluorescence emission measurements and determined using Equation (1). Three independent measurements were performed for each system, and standard deviations (SD) were calculated.

(1) $$EE\left(\%\right)=\frac{\left(\mathrm{total}\;\mathrm{concentration}-\mathrm{concentration}\;\mathrm{of}\;\text{non-encapsulated}\;\mathrm{drug}\right)}{\mathrm{total}\;\mathrm{concentration}}\times 100$$

After preparation, drug-loaded liposomes were subjected to centrifugation at 3000 rpm for 10 min in Amicon^®^ Ultra Centrifugal Filters (10 kDa, Merck, Darmstadt, Germany), with 2.5 mL of the sample in the upper compartment and 5 mL of the phosphate buffer in the bottom (volumes determined by the parameter/sample volume of Amicon^®^ filters). Here, the retentate contains the drug-loaded liposomes, and the filtrate contains the non-encapsulated compound. After centrifugation, the filtrate was pipetted out, and its volume was measured and evaporated at 80 °C. The evaporated volume of filtrate was replaced by the same amount of ethanol, and its fluorescence spectrum was measured. The maximum fluorescence intensity of the filtrate allowed the concentration of the non-encapsulated drug, using the calibration curve of fluorescence intensity vs. concentration previously obtained (Figure S1 in Supplementary Materials), to be determined.

##### Drug Release Assays

The release assays of the antitumor compound from the different nanoformulations were performed on PBS buffer, using dialysis membranes (10K MWCO, ThermoFisher Scientific, Waltham, MA, USA), with 1 mL of liposomes inside the dialysis bag and 4 mL of buffer at the outside. The assay was performed with stirring in an orbital shaker RSLAB-7, at room temperature. The concentration of the released drug was measured at different times, for 72 or 79 h. At the different time points, 200 µL aliquots were collected, and an equal volume of fresh buffer was added to determine the cumulative compound release. The fluorescence intensity of the several aliquots was measured, allowing the corresponding concentration to be determined.

The release profiles were fitted to a first-order kinetic model and to the Weibull model:

The first-order kinetic model [66] follows Equation (2),

(2) $$F\;\left(\%\right)=M_{0}\times (1-e^{-kt})$$

where *F* (%) is the percentage of the released compound, *M*_0_ represents the total amount of compound released, *k* represents the first-order rate constant, and *t* is the time.

The Weibull model [67] is a distribution function, which expresses the compound fraction accumulated, where $M_{t}$ and $M_{\infty }$ are the cumulative amounts of drug released at time *t* and infinite time, respectively, in solution, following Equation (3),

(3) $$\frac{M_{t}\;}{M_{\infty }}=1-\exp\left[-{at}^{b}\right]$$

where *a* is a parameter defining the timescale of the process and *b* denotes the curve type shape parameter. For *b* > 1, the transport follows a complex release mechanism; *b* ≤ 0.75 indicates Fickian diffusion (in either fractal or Euclidian spaces), and 0.75 < *b* < 1 indicates a combined mechanism (Fickian diffusion and Case II transport) [67].

### 3.3. Anticancer Activity

#### 3.3.1. Cell Culture and Buffer Solutions

The colorectal cancer cell line HCT 116 (ATCC CCL-247) was grown in Roswell Park Memorial Institute 1640 Medium (RPMI, Biochrom Ltd., Cambridge, UK), and the breast cancer cell line MDA-MB-231 (ATCC HTB-26, American Type Culture Collection, Manassas, VA, USA) was maintained in Dulbecco’s Modified Eagle Medium (DMEM, Biochrom Ltd., Cambridge, UK) supplemented with 10% (*v*/*v*) fetal bovine serum (FBS, Biochrom Ltd., Cambridge, UK) and 1% (*v*/*v*) penicillin-streptomycin (p/s, Biochrom) on tissue-culture-treated flasks. The human fibroblast BJ-5ta cell line (ATCC CRL-2522) was grown in a 4:1 proportion of DMEM:Medium 199 (PAN-Biotech GmbH, Aidenbach, Germany), supplemented with 10% (*v*/*v*) FBS, 1% (*v*/*v*) p/s, and 0.01% (*v*/*v*) hygromycin B (Sigma-Aldrich, St. Louis, MO, USA). All cell lines were authenticated and tested for mycoplasma contamination. Cells were incubated at 37 °C with 5% CO_2_ and 95% relative humidity. The cells were washed using phosphate-buffered saline (PBS 1×, pH 7.4) and were detached using Trypsin/EDTA (Biochrom). Cell counts were determined using a hemocytometer.

For MTT experiments, logarithmically growing HCT 116 and MDA-MB-231 cells were seeded in 96-well plates at a concentration of 0.5 × 10^5^ cells/mL, and 0.8 × 10^5^ cells/mL for BJ-5ta cells. For uptake experiments, MDA-MB-231 cells were seeded in 6-well plates at 1 × 10^5^ cells/mL.

#### 3.3.2. Cytotoxicity Assays

To study the cytotoxic effect of the compounds and determine their IC_50_, MDA-MB-231, HCT 116, and BJ-5ta cells were plated and incubated at 37 °C with 5% CO_2_ for 24 h. In each independent experiment, **PP** (2 mM) and 5-FU (20 mM) stock solutions were prepared in DMSO. HCT 116 cells were treated with **PP** concentrations (0.5, 1, 2, 3, 6, and 10 µM) or 5-FU (5, 10, and 15 µM) in 100 µL of culture medium at 37 °C for 48 h. MDA-MB-231 cells were exposed to different **PP** concentrations (0.5, 1.5, 2.5, 5, 10, and 15 µM) or 5-FU (80, 100, 150, and 200 µM) under the same conditions. A control group of cells received DMSO (1%) (*v*/*v*).

To evaluate the effects of placebo liposomal nanoformulations (**DPPC-L**, **DPPC-Chol-L**, **EggPC-L**, and **EggPC-Chol-L**), cells were exposed to different lipid concentrations (dissolved in 100 µL of culture medium) at 37 °C for 48 h. HCT 116 cells were treated with 0.190 or 0.375 mM, MDA-MB-231 cells with 0.314 or 0.943 mM, and BJ-5ta cells with 0.190 and 0.943 mM. For these assays, stock solutions of 5 mM in lipid were used.

For **PP**-loaded liposomal nanoformulations (**PP-DPPC-L**, **PP-DPPC-Chol-L**, **PP-EggPC-L**, and **PP-EggPC-Chol-L**), the three cell lines were treated with **PP** at different concentrations, alongside 1% DMSO (control), in 100 µL of culture medium at 37 °C for 48 h. HCT 116 cells were exposed to **PP** at IC_50_ (2 µM) and 2-fold IC_50_ concentrations (4 µM). MDA-MB-231 cells received **PP** at IC_50_ (5 µM) and 3-fold IC_50_ (15 µM). BJ-5ta cells were exposed to **PP** at 3 and 15 µM.

After the incubation period, the culture media was discarded from each well, and 100 µL (0.5 mg/mL) was added. The plates were incubated for 2 h, after which the supernatant was removed, and 100 µL of DMSO was added to dissolve purple formazan crystals for 15 min at room temperature (RT). Cell viability was determined based on the absorbance measured at 570 nm using a microplate reader (Cytation 3, BioTek Instruments, Winooski, VT, USA). The absorbance of untreated cells or DMSO-treated cells was considered at 100% cell viability. Triplicate assays were performed, with all conditions tested in at least triplicate wells.

#### 3.3.3. Uptake Experiments

After cell seeding for 24 h, MDA-MB-231 cells were incubated with CM-DiI-labelled liposomal formulations (0.943 mM of lipid) for 3, 6, and 24 h. After that, the medium was discarded, the wells were washed with PBS 1×, and trypsin was added to detach the cells. Flow cytometry analysis, with a BD LSR Fortessa X-20 equipment (BD Biosciences, Heidelberg, Germany), was used to determine the percentage of cells that internalized labelled liposomal formulations, with data analyzed using FCS Express 6 Flow Research edition software.

For microscopy, cells grown on coverslips were fixed with 4% PFA for 1 h at RT, washed with PBS 1×, and incubated with NH_4_Cl, PBS-SDS (0.1%), and BSA (3%), for 10 to 20 min, with PBS 1× rinses in between. Cells were then exposed to Alexa Fluor 488-Phalloidin for 1 h in the dark, followed by PBS 1× rinsing and DAPI incubation for 15 min at RT. Cells were washed, coverslips were mounted, and images were captured using a fluorescence microscope (Olympus BX51, Hachioji, Tokyo, Japan) incorporated with a high-sensitivity camera Olympus DP71 (Olympus, Hachioji, Tokyo, Japan), using a 40× objective and with the precise filter settings for CM-DiI, Alexa Fluor 488 and DAPI.

### 3.4. Statistical Analysis

For each assay at least three independent experiments were conducted. Results are presented as mean ± standard mean of error (SEM) or SD (standard deviation). The inhibitory concentration in half-maximal inhibitory concentration (IC_50_) was calculated using GraphPad Prism 8 (GraphPad Software, Inc., La Jolla, CA, USA). GraphPad Prism was utilized to produce a graphical representation of the results, and statistical significance was analyzed by one-way analysis of variance (ANOVA) to compare control vs. experimental groups, or between experimental groups. Statistical significance was established for all comparisons, at significance levels of ns (non-statistical significance) *p* > 0.05, * *p* ≤ 0.05, ** *p* ≤ 0.01, *** *p* < 0.001, and **** *p* < 0.0001.

## 4. Conclusions

A promising pyrimido[5,4-*d*]pyrimidine compound (**PP**) was synthesized in excellent yields and incorporated in liposomal nanoformulations based on PC. The **PP**-loaded liposomes (**PP-DPPC-L**, **PP-EggPC-L**, **PP-EggPC-Chol-L**, and **PP-DPPC-Chol-L**) exhibited hydrodynamic diameters of less than 150 nm and low polydispersity, together with a good stability at 4 °C. The **PP**-loaded liposomal formulations were obtained with excellent encapsulation efficiencies (>95%) and displayed a sustained release, matching a Fickian diffusion mechanism.

The compound **PP** showed potent anticancer activity, with IC_50_ values of 2.04 ± 0.45 µM and 5.24 ± 0.24 µM for HCT116 and MDA-MB-231 cell lines, respectively. When encapsulated in liposomal formulations, the anticancer activity of **PP** was largely preserved in most cases. However, a slightly lower activity was observed for **PP-EggPC-L**, which is hypothesized to be due to its higher fluidity. Efficient cellular uptake of **PP-EggPC-L** and **PP-EggPC-Chol-L** into MDA-MB-231 cells was confirmed.

While **PP-DPPC-L** and **PP-DPPC-Chol-L** liposomes exhibited anticancer activity similar to the free drug, they also presented toxicity against the non-tumor BJ-5ta cell line. On the other hand, the **PP-EggPC-Chol-L** formulation revealed very low toxicity against normal cells and notable anticancer activity for both cancer cell lines, suggesting it is a suitable candidate for in vivo assays.

## Figures and Tables

**Table 1:** Hydrodynamic size, polydispersity (PDI), and zeta potential of drug-loaded liposomal formulations (**PP-EggPC-L**, **PP-DPPC-L**, **PP-EggPC-Chol-L**, and **PP-DPPC-Chol-L**) and non-loaded liposomal formulations (**EggPC-L**, **DPPC-L**, **EggPC-Chol-L**, and **DPPC-Chol-L**). SD: standard deviation.

Liposomal Formulations	Hydrodynamic Diameter ± SD (nm)	PDI ± SD	Zeta Potential ± SD (mV)
**EggPC-L**	145 ± 7	0.26 ± 0.02	−0.4 ± 0.1
**PP-EggPC-L**	142 ± 27	0.25 ± 0.01	−2.2 ± 1.5
**DPPC-L**	104.1 ± 2.2	0.26 ± 0.02	1.1 ± 0.3
**PP-DPPC-L**	104 ± 15	0.25 ± 0.01	1.1 ± 1.3
**EggPC-Chol-L**	147.8 ± 7	0.25 ± 0.01	−2.9 ± 0.4
**PP-EggPC-Chol-L**	142.6 ± 4	0.25 ± 0.01	−3.9 ± 0.3
**DPPC-Chol-L**	100.9 ± 3	0.25 ± 0.01	1.8 ± 0.3
**PP-DPPC-Chol-L**	99.2 ± 4.6	0.20 ± 0.01	2.3 ± 0.1

**Table 2:** Hydrodynamic size and polydispersity (PDI) of nanoformulations after lyophilization. SD: standard deviation of the several assays.

Liposomal Nanoformulations	Hydrodynamic Diameter ± SD (nm)	PDI ± SD
**PP-EggPC-L**	134 ± 16	0.26 ± 0.01
**PP-EggPC-Chol-L**	222 ± 32	0.27 ± 0.01
**PP-DPPC-L**	147 ± 20	0.26 ± 0.02
**PP-DPPC-Chol-L**	88 ± 25	0.23 ± 0.06

**Table 3:** Drug encapsulation efficiencies.

Liposomal Formulation	Lipid	EE (%)
**PP-EggPC-L**	Egg-PC	96 ± 3
**PP-DPPC-L**	DPPC	99.7 ± 0.1
**PP-EggPC-Chol-L**	Egg:Cholesterol (7:3)	99.1 ± 0.1
**PP-DPPC-Chol-L**	DPPC:Cholesterol (7:3)	99.5 ± 0.4

## Data Availability

Data presented in this study is contained within the article and Supplementary Material. Further inquiries can be directed to the corresponding authors.

## Supplementary Materials

The following supporting information can be downloaded at https://www.mdpi.com/article/10.3390/ph18081210/s1, (S1) Materials and Methods: (S1.1) ^1^H and ^13^C NMR spectra of compounds and (S1.2) anticancer activity; (S2) Results: (S2.1) **PP** spectra and calibration curve of fluorescence intensity vs. concentration: Figure S1: Absorption and fluorescence emission spectra of **PP** compound (10^−6^ M in ethanol) (A) and calibration curve of fluorescence intensity of **PP** vs. concentration (B); (S2.2) Fitting of drug release profiles: Table S1: Parameters obtained by fitting the release profiles to the first-order kinetic model and Weibull model and the respective coefficients of determination (R^2^); (S2.3) Biological assays: Figure S2: Assessment of the viability of HCT 116 (A) and MDA-MB-231 (B) cancer cells after 48 h exposure to 5-FU; Figure S3: Assessment of the viability of HCT 116 (A), MDA-MB-231 (B), and BJ-5ta (C) after 48 h exposure to placebo liposomal nanoformulations.

## Abbreviations

The following abbreviations are used in this manuscript:

PP	Pyrimido[5,4-*d*]pyrimidine compound
5-FU	5-Fluorouracil
DBU	1,8-Diazabicyclo[5.4.0]undec-7-ene
L	Liposomes
PC	Phosphatidylcholine
EggPC	Egg yolk
DPPC	Dipalmitoylphosphatidylcholine
Tc	Transition temperature
Chol	Cholesterol
PDI	Polydispersity
DLS	Dynamic light scattering
EE	Encapsulation efficiency
PBS	Phosphate-buffered saline
