# Supplementary material for: Liposomal Formulations for Efficient Delivery of a Novel, Highly Potent Pyrimidine-Based Anticancer Drug
Source: Pharmaceuticals (Basel). 2025 Aug 15;18(8):1210. doi: 10.3390/ph18081210 (PMC12389155; doi:10.3390/ph18081210)
Supplement: Supplementary file 1 [file pharmaceuticals-18-01210-s001.zip › pharmaceuticals-3816730-supplementary.pdf]

Electronic Supplementary Information (ESI)

Liposomal Formulations for Efficient Delivery of a Novel Highly Potent Pyrimidine-Based Anticancer Drug

Sofia Teixeira [1,2], Débora Ferreira [3,4], Ana Rita O. Rodrigues [2], Ligia R. Rodrigues [3,4], Elisabete M. S. Castanheira [2*], M. Alice Carvalho [1,*]

[1] Chemistry Centre of University of Minho (CQ-UM), Campus de Gualtar, 4710-057 Braga, Portugal; id9191@uminho.pt (S.T.); mac@quimica.uminho.pt (M.A.C.)

[2] Physics Centre of Minho and Porto Universities (CF-UM-UP) and LaPMET (Laboratory of Physics for Materials and Emergent Technologies), University of Minho, Campus de Gualtar, 4710-057 Braga, Portugal; ecoutinho@fisica.uminho.pt (E.M.S.C); ritarodrigues@fisica.uminho.pt (A.R.O.R)

[3] Centre of Biological Engineering (CEB), University of Minho, Gualtar, 4710-057 Braga, Portugal; lrmr@deb.uminho.pt (L.R.R.); deboraferreira@ceb.uminho.pt (D.F.)

[4] LABBELS – Associated Laboratory, Braga, Guimarães, Portugal; lrmr@deb.uminho.pt (L.R.R.); deboraferreira@ceb.uminho.pt (D.F.)

* Correspondence: ecoutinho@fisica.uminho.pt (E.M.S.C); mac@quimica.uminho.pt (M.A.C.)

Index

1. Materials and methods 2
1.1. ^{1}H and ^{13}C NMR spectra of compounds 2
1.2. Anticancer activity 4
1.2.1. Reagents 4
2. Results 4
2.1. PP spectra and calibration curve of fluorescence intensity vs. concentration 4
2.2. Fitting of drug release profiles 4
2.3. Biological assays 5

1. Materials and methods

1.1. ¹H and ¹³C NMR spectra of compounds

N-(4-imino-8-((4-(trifluoromethoxy)phenyl)amino)pyrimido[5,4-*d*]pyrimidin-3(4*H*)-yl)benzamide **9**

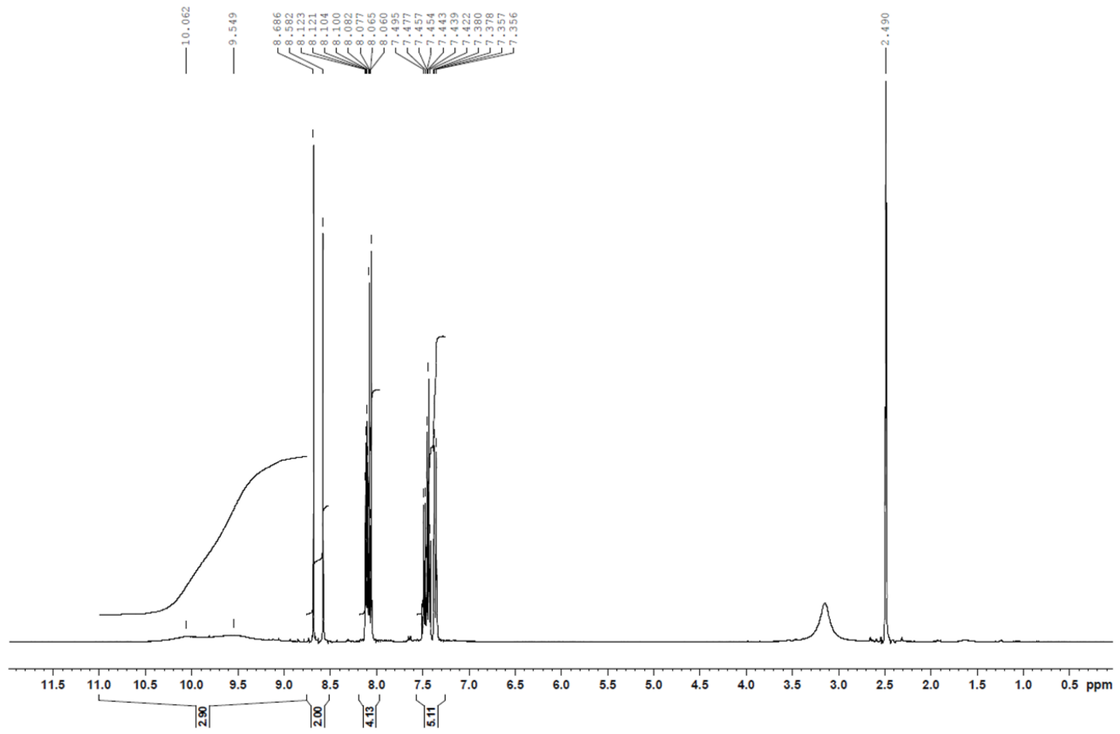

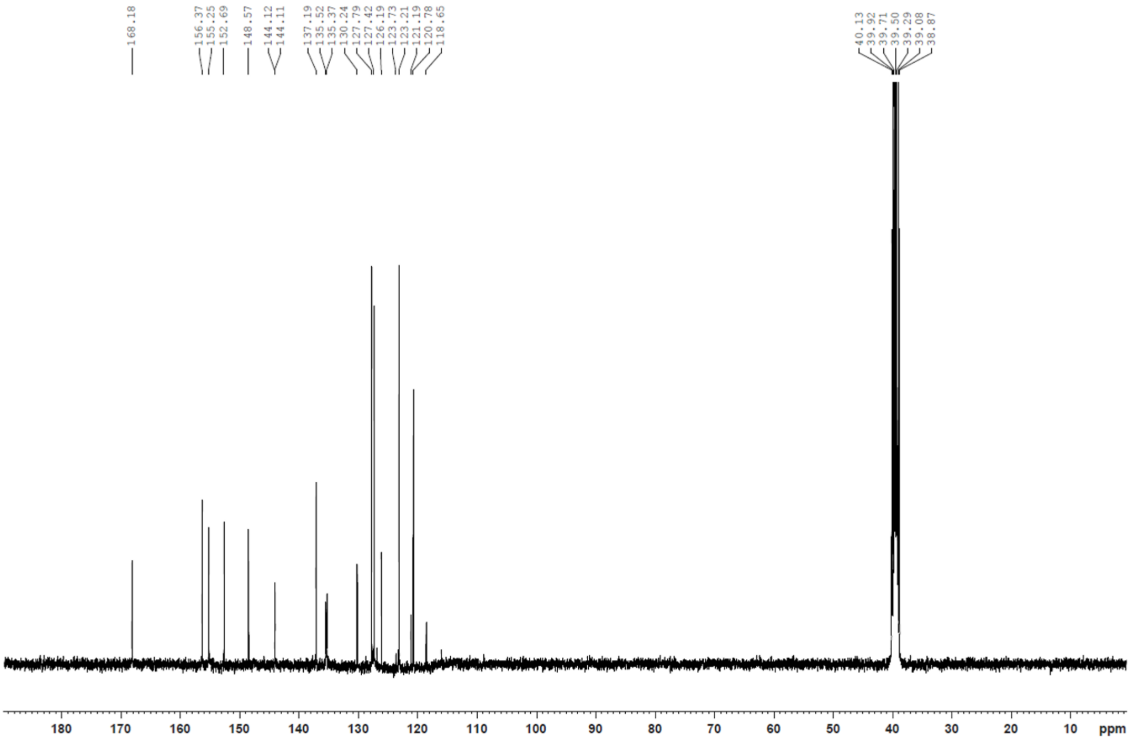

N'-(8-((4-(trifluoromethoxy)phenyl)amino)pyrimido[5,4-*d*]pyrimidin-4-yl)benzohydrazide **PP**

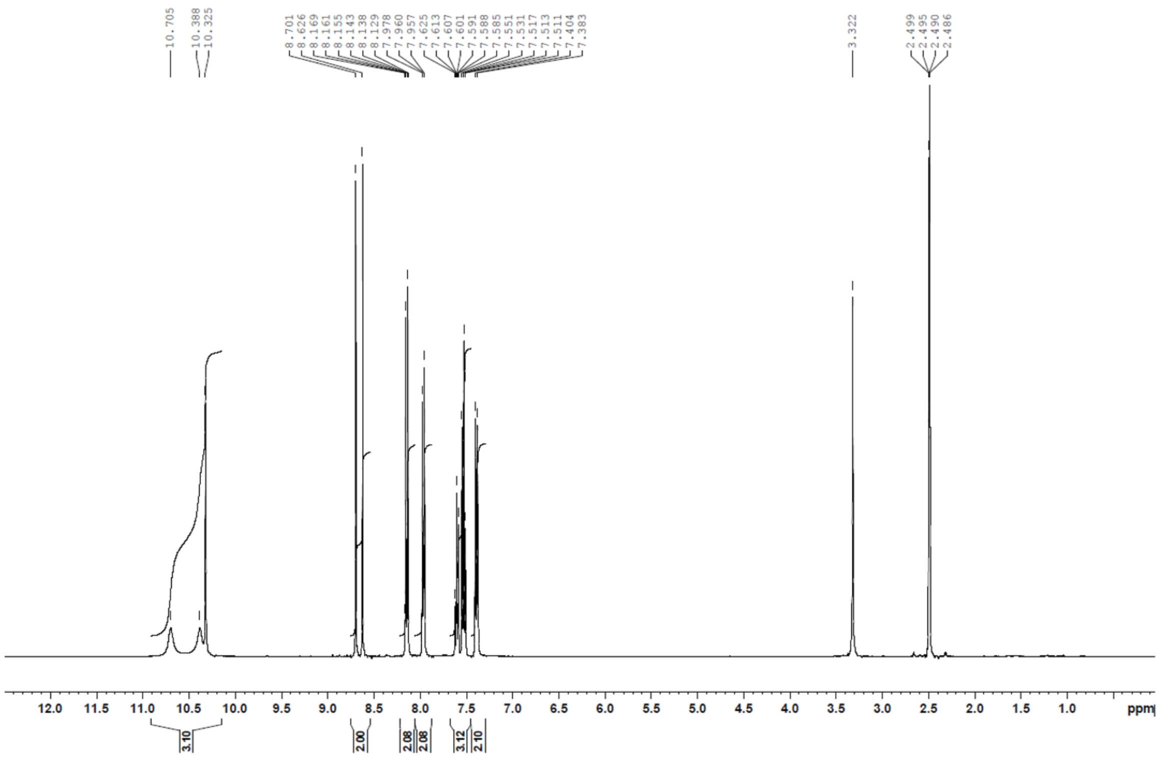

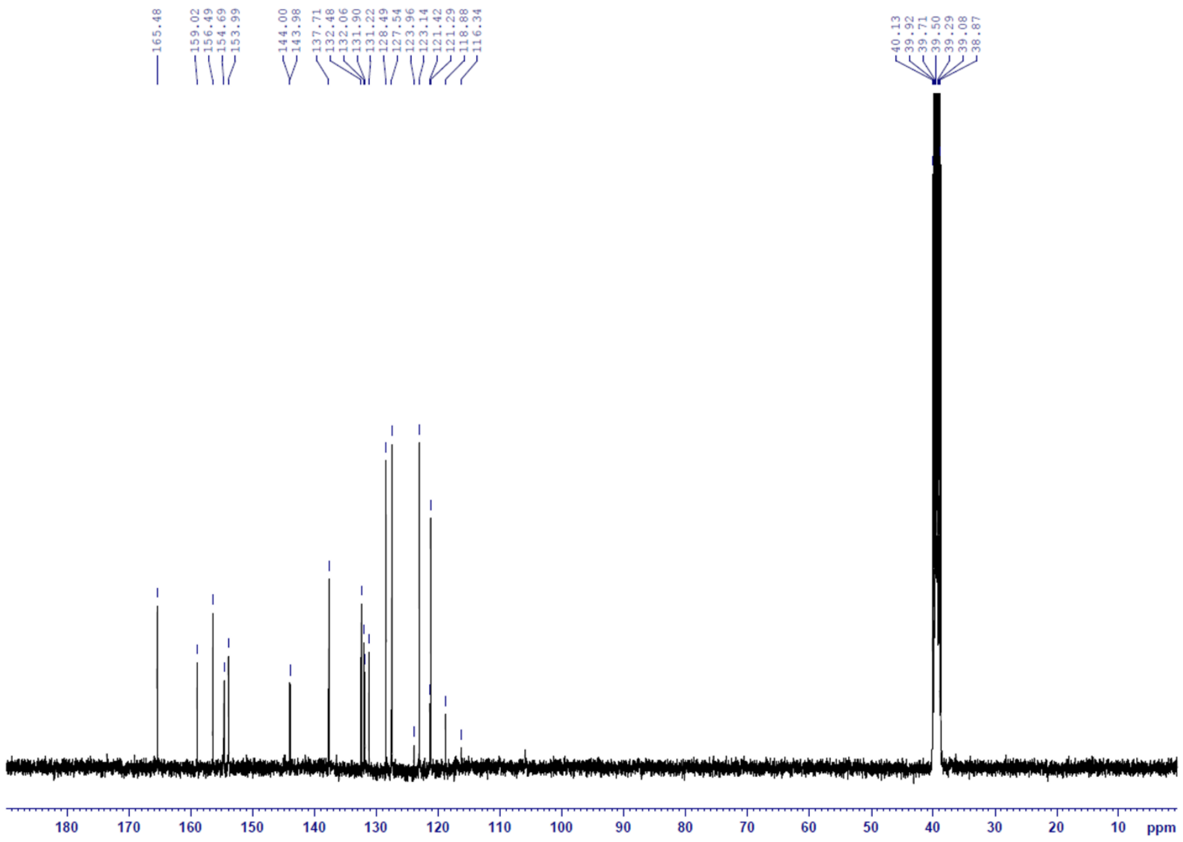

1.2. Anticancer activity

1.2.1. Reagents

3-(4,5-Dimethylthiazol-2-yl)-2,5-Diphenyltetrazolium Bromide (MTT) reagent and paraformaldehyde (PFA) were acquired from Sigma-Aldrich. 4′,6-Diamidino-2-Phenylindole dihydrochloride (DAPI) and 1,1′- -Dioctadecyl-3,3,3′,3′-Tetramethylindocarbocyanine Perchlorate (CM-DiI Dye) were acquired from Biotium and Invitrogen, respectively. Alexa Fluor 488-Phalloidin was acquired from Molecular Probes. Bovine Serum Albumin (BSA) was purchased from Nzytech. Sodium dodecyl sulfate (SDS) and dimethyl sulfoxide (DMSO) were obtained from Thermo Fisher Scientific.

2. Results

2.1. ***PP*** *spectra and calibration curve of fluorescence intensity vs. concentration*

Figure S1 shows the absorption and fluorescence spectra of the novel **PP** compound and the calibration curve of fluorescence intensity *vs.* concentration.

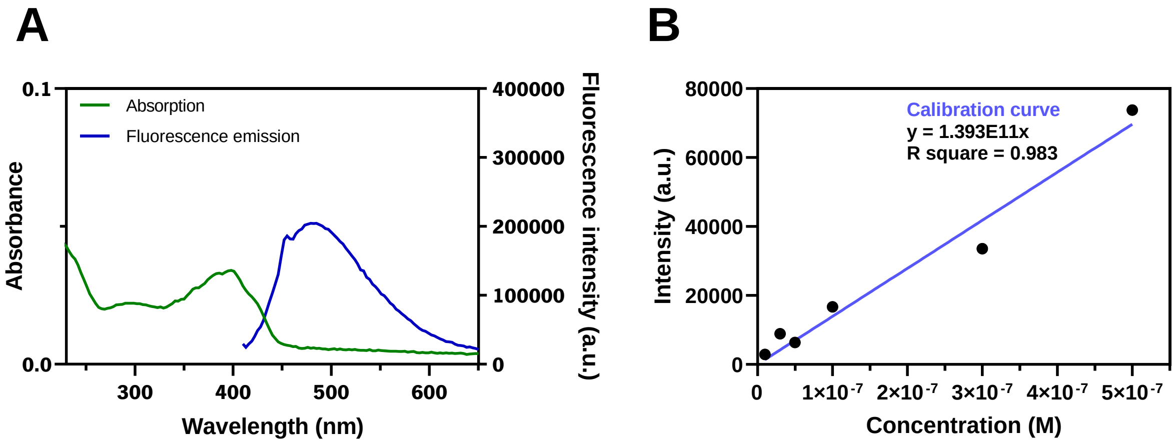

Figure S1. Absorption and fluorescence emission spectra of **PP** compound (10^{-6} M in ethanol) (**A**) and calibration curve of fluorescence intensity of **PP** vs. concentration (**B**).

2.2. Fitting of drug release profiles

The results of the fitting of release profiles to first-order kinetics and Weibull models are displayed in **Table S1**.

Table S1. Parameters obtained by fitting the release profiles to the first-order kinetic model and Weibull model, and the respective coefficients of determination (R^2).

Liposomal formulations	First-order kinetics		Weibull		
	k (min^{-1})	R^2	b	a	R^2
**PP-EggPC-L**	0.004009	0.914	0.5928	0.0326	0.963
**PP-EggPC-Chol-L**	0.003876	0.720	0.2954	0.04751	0.968
**PP-DPPC-L**	0.004873	0.697	0.3419	0.0841	0.944
**PP-DPPC-Chol-L**	0.003978	0.788	0.3691	0.06816	0.971

2.3. Biological assays

Figure S2 shows that the viability of HCT 116 and MDA-MB-231 cell lines decreased with increasing concentrations of 5-fluorouracil (**5-FU**), demonstrating a dose-dependent effect. **5-FU** showed IC_{50} values of 10.39 ± 0.68 μM for HCT 116 and 183.49 ± 17.13 μM for MDA-MB-231 cells.

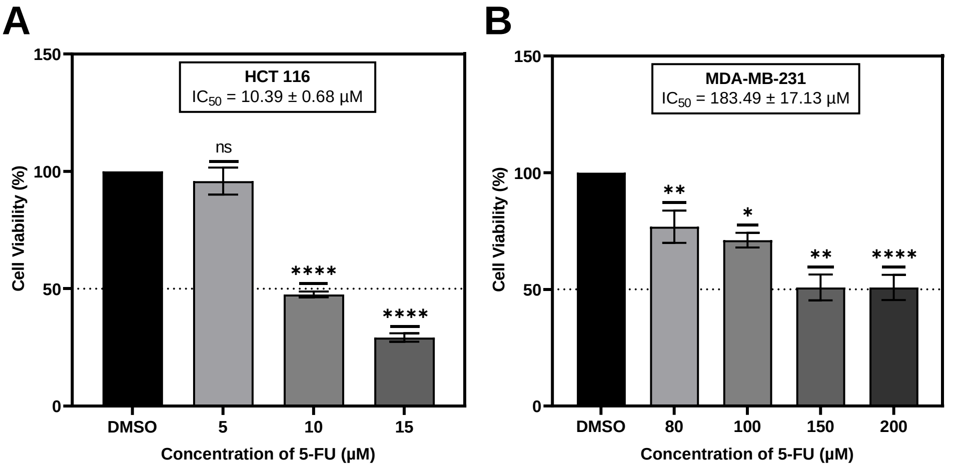

Figure S2. Assessment of the viability of HCT 116 (**A**) and MDA-MB-231 (**B**) cancer cells after 48 hours exposure to **5-FU**. HCT 116 **(A)** exposure to **5-FU** concentrations of 5, 10 and 15 μM over a 48-hour treatment period. **MDA-MB-231 (B, D)** exposure to **5-FU** concentrations of 80, 100, 150 and 200 μM over a 48-hour treatment period. Cell viability was determined using the MTT colorimetric assay, and normalized to DMSO treated cells. The resulting data are presented as IC_{50} (in μM) ± SEM (standard error of the mean) from three independent experiments. One-way ANOVA indicates statistically significant differences to DMSO assessed by Dunnett's post-test, and denoted as follows: ns (non-statistical significance), * $p \leq 0.05$, ** $p \leq 0.01$, *** $p < 0.001$, and **** $p < 0.0001$.

Figure S3 shows the cytotoxic effect of placebo nanoformulations against HCT 116 and MDA-MB-231 cancer cells and BJ-5ta normal cells.

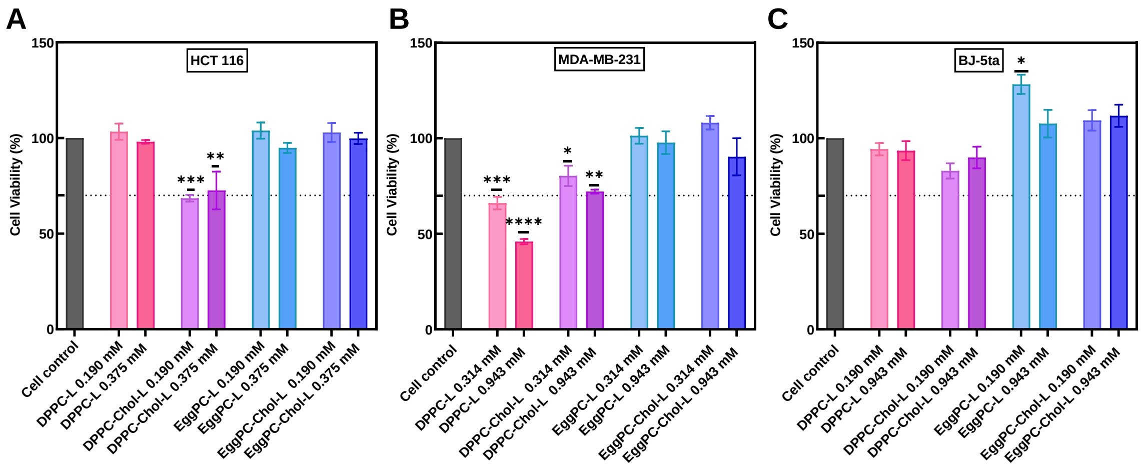

Figure S3. Assessment of the viability of HCT 116 (**A**), MDA-MB-231 (**B**) and BJ-5ta (**C**) after 48 hours exposure to placebo liposomal nanoformulations. HCT 116 (**A**) exposure to placebo liposomal nanoformulations concentrations of 0.190 and 0.375 mM, over a 48-hour treatment period. MDA-MB-231 (**B**) exposure to placebo liposomal nanoformulations concentrations of 0.314 and 0.943 mM, over a 48-hour treatment period. BJ-5ta (**C**) exposure to placebo liposomal nanoformulations concentrations of 0.190 and 0.943 mM, over a 48-hour treatment period. Cell viability was determined using the MTT colorimetric assay, and normalized to untreated cells. One-way ANOVA indicates statistically significant differences to control by Dunnett's post-test, and denoted as follows: * $p \leq 0.05$, ** $p \leq 0.01$, *** $p < 0.001$, and **** $p < 0.0001$.
